# Draft Genome Sequences of Symbiotic and Nonsymbiotic Rhizopus microsporus Strains CBS 344.29 and ATCC 62417

**DOI:** 10.1128/genomeA.01370-14

**Published:** 2015-01-22

**Authors:** Fabian Horn, Zerrin Üzüm, Nadine Möbius, Reinhard Guthke, Jörg Linde, Christian Hertweck

**Affiliations:** a Systems Biology/Bioinformatics, Leibniz Institute for Natural Product Research and Infection Biology - Hans Knöll Institute, Jena, Germany; b Biomolecular Chemistry, Leibniz Institute for Natural Product Research and Infection Biology - Hans Knöll Institute, Jena, Germany

## Abstract

Specific *Rhizopus* microsporus pathovars harbor bacterial endosymbionts (Burkholderia rhizoxinica) for the production of a phytotoxin. Here, we present the draft genome sequences of two R. microsporus strains, one symbiotic (ATCC 62417), and one endosymbiont-free (CBS 344.29). The gene predictions were supported by RNA sequencing (RNA-seq) data. The functional annotation sets the basis for comparative analyses.

## GENOME ANNOUNCEMENT

The zygomycete *Rhizopus* microsporus is a terrestrial filamentous fungus that is used in food fermentation but also is known to cause rice seedling blight, which leads to substantial losses in agriculture, as well as mucormycoses in immunocompromised patients (1, 2). Phytopathogens, such as R. microsporus strain ATCC 62417, harbor endosymbiotic bacteria of the genus Burkholderia (3, 4) producing the antimitotic agent rhizoxin (5–7). Surprisingly, these symbiotic fungi lose their ability to produce mature sporangia and vegetative spores in the absence of the endobacteria (8). An analysis of the genome sequence of the endosymbiont Burkholderia rhizoxinica has revealed bacterial symbiosis factors (9–12). Besides information gained from genetic bar code studies (13), nothing is known about the genomic differences between symbiotic and symbiont-free R. microsporus strains.

For each strain, multiple libraries (paired-end, 2-kb mate-pair, 3 × 5-kb mate-pair) were sequenced with Illumina HiSeq 2000. R. microsporus CBS 344.29 was additionally sequenced using Illumina MiSeq V2. The reads were quality trimmed, error corrected (14), digitally normalized (15), and finally assembled with AllPaths-LG (16) (LGC Genomics, Berlin, Germany). The assemblies were postprocessed using SOAP GapCloser (17). Transcriptome sequencing was performed using HiSeq 2000 100 bp. CBS 344.29 was cultured on potato dextrose agar (PDA) plates for 3 days at 30°C. ATCC 62417 was cultured under three different conditions, namely, with and without the endosymbiont (B. rhizoxinica) on PDA plates, and with bacterium in Vorkultur medium at 30°C for 3 days. The culture with Vorkultur medium was sequenced using the 454 FLX Titanium platform, and the contigs were assembled using Newbler (454 Life Sciences). rRNA was detected and removed with riboPicker (18).

Structural and functional gene annotation were performed as described previously (19, 20). Protein sequences from *Lichtheimia hyalospora* (Joint Genome Institute [JGI]), *Mucor circinelloides* (Broad Institute), *Phycomyces blakesleeanus* (JGI), *Rhizopus delemar* (Broad Institute), and R. microsporus var. microsporus (JGI) were mapped to the reference genomes. The transcriptome assemblies of Newbler, Cufflinks (21), and genome-independent and genome-guided Trinity (22) were combined using EvidentialGene (23). The resulting assembly was checked for contamination with the help of BLAST, the NCBI nonredundant database, and the UniProt fungal knowledge base.

The genome assembly is based on sequencing data amounting to 15.9 Gbp (CBS 344.29) and 15.7 Gbp (ATCC 62417), which represent a 310-fold estimated genome coverage. The assembly for CBS 344.29 consists of 1,554 scaffolds, with a total size of 49.2 Mbp (*N*_50_, 53 kbp), whereas the assembly of ATCC 62417 consists of 1,386 scaffolds, with a total size of 49.6 Mbp (*N*_50_, 198 kbp). The exceptionally low G+C content of the assemblies are 34.5% and 33.4%, respectively. Using CEGMA (24), we identified 431 and 451 core proteins within the respective genomes. During the genome annotation, we utilized RNA sequencing (RNA-seq) data amounting to 5.5 Mbp (CBS 344.29) and 5.1 Mbp (ATCC 62417), which represent an estimated 100-fold transcriptome coverage. For CBS 344.29, we predicted 19,564 genes and 20,209 transcripts, of which 10,698 have GO categories, 14,642 have InterPro domains, and 3,879 contain transmembrane domains. For ATCC 62417, we predicted 18,869 genes and 23,603 transcripts, of which 9,704 have GO categories, 17,264 have InterPro domains, and 4,807 contain transmembrane domains. The GO annotations of the fungus have been made available for enrichment analysis with FungiFun2 (25).

### Nucleotide sequence accession numbers.

This whole-genome shotgun project has been deposited in DDBJ/ENA/GenBank under the accession numbers CCYT01000001 to CCYT01001386 (strain ATCC 62417) and CDGI01000001 to CDGI01001554 (strain CBS 344.29). The versions described in this paper are the first versions. The genome data and additional information are also available at the HKI Genome Resource (http://www.genome-resource.de/).
